# Feeling Seen, Being Heard: Perspectives of Patients Suffering from Mental Illness on the Possibility of Physician-Assisted Death in the Netherlands

**DOI:** 10.1007/s11013-021-09726-5

**Published:** 2021-06-16

**Authors:** Rosalie Pronk, Dick L. Willems, Suzanne van de Vathorst

**Affiliations:** 1 Department of Ethics, Law and Humanities, Amsterdam UMC, Academic Medical Centre, Room J2-126, PO Box 22660, 1100 DD Amsterdam, The Netherlands; 2 Department of Medical Ethics and Philosophy, Erasmus Medical Centre, Rotterdam, The Netherlands

**Keywords:** Physician-assisted dying, euthanasia, mental illness, Netherlands, psychiatry

## Abstract

Physician-assisted death (PAD) for patients suffering from a mental illness is allowed in the Netherlands under certain conditions but is a very controversial topic, mainly discussed by ethicists and physicians. The voice of the patient is rarely included in the debate, so we know little about what their views on the topic are. We aim to understand the views of patients with mental illness and wish to die with regard to the possibility of PAD in the Netherlands. The data for this qualitative study were collected through 21 in-depth interviews with Dutch patients who have a wish for PAD as a result of suffering from a mental illness. We identified four themes in relation to the meaning of PAD for the patients suffering from mental illness and wish to die. These themes are (1) Autonomy and self-determination, (2) ending the suffering, (3) recognition, and (4) a dignified end-of-life. The option of PAD for patients suffering from mental illnesses was considered of great importance to the patients who have a wish to die. We highlight the importance of ‘recognition’ for the situation of the patient, as this could lead to new perspective. We argue that psychiatrists need to reflect on providing this recognition in earlier phases of treatment, taking seriously and discussing a wish for PAD in treatment is beneficial to patients. It provides space for the patient to discuss their wishes and could cause them not wanting to die anymore.

## Introduction

Physician-assisted death (PAD) remains a very controversial topic (the term ‘Medical Aid in Dying’ (MAID) is also used, predominantly in Canada and the US). Few countries in the world allow PAD under certain conditions, and the Netherlands is one of these countries. The Dutch euthanasia law has regulated PAD since 2002 and allowed for PAD in case of a medical condition and if physicians act in compliance with the criteria of due care. These criteria can be found in Box 1. Patients suffering from mental illness are not excluded from eligibility for PAD, if their physician can comply with the due care criteria. Since the enactment of the law, the number of patients that received PAD based on suffering from mental illness is small but increasing. Before 2008, mental illness was not registered as a separate category, but from 2008 to 2019, the number of cases rose from 0 to 68 (with a peak of 83 in 2017) (Regionale Toetsingscommissie Euthanasie^2019^).

**Box 1:** Legal criteria of due care

1. The physician must be satisfied that the patient has made a voluntary and carefully considered request; 2. The physician must be satisfied that the patient’s suffering is unbearable, and that there is no prospect of improvement; 3. The physician must have informed the patient about his situation and his prospects; 4. The physician must have come to the conclusion, together with the patient that there is no reasonable alternative in the light of the patient’s situation; 5. The physician must have consulted at least one other, independent physician, who must have seen the patient and given a written opinion on the due care criteria referred to above; 6. And the physician must terminate the patient’s life or provide assistance with suicide with due medical care and attention.	

Dutch physicians are reluctant to perform PAD in case of mental illness (more so than for PAD in case of somatic conditions), and psychiatrists are becoming more reluctant over the years (Onwuteaka-Philipsen et al. 2017; Evenblij et al. 2019). Most requests are received and performed by Expertise Centre Euthanasia (formerly known as End-of-Life Clinic) (Onwuteaka-Philipsen et al. 2017; Kammeraat and Kolling 2020). To guide physicians, various guidelines were drafted to provide clarification on how to handle a request for PAD from patients suffering from a mental illness, and on how to interpret the criteria of due care. The Regional Review Committees (RTE) and the Dutch Association for Psychiatry both issued guidelines on how to deal with a PAD request by a patient suffering from mental illness (Levensbeëindiging op verzoek bij patiënten met een psychische stoornis [Internet] 2018; Regionale Toetsingscommissie Euthanasie 2018). These guidelines state that in these cases, a ‘second opinion’ should be given by a physician specialized in the condition of the patient, over and above the second-opinion already mandatory by law (SCEN consult).

Points of discussion in the debate include the difficulty of interpreting the criteria of due care in case of mental illness, the complexity of establishing whether the death wish is part of the psychiatric disorder (Miller and Appelbaum 2018; Kim and Lemmens 2016), the assessment of mental capacity (Evenblij 2019), irrational beliefs held by the patient about chances of recovery (Kim and Lemmens 2016; Blikshavn et al. 2017), the vulnerability of psychiatric patients, their need for protection (Kim and Lemmens 2016; Lemmens 2016; Rooney et al. 2017), and the concept of hope in treatment (Berghmans et al. 2013; Kissane and Kelly 2000). While scholars discuss these issues, the voice of the patient is rarely heard (Verhofstadt et al. 2017). Therefore, we set up a study that investigates the views of patients suffering from mental illness, who have a wish for PAD.

## Methods

We held 21 in-depth interviews with patients who suffer from mental illness and who have a wish for PAD. These interviews were conducted between November 2019 and July 2020. We used a topic list that can be found in Box 2.

**Box 2:** Topic list

- What is the respondent’s situation? - Why do they have a wish to die, how do they experience having a wish to die? - What makes their suffering so unbearable that they wish for PAD? - Why do they believe their suffering is without prospect of improvement? - What does the option of PAD mean to them? - How did they experience the process of requesting PAD? - How did they feel about how the physician handled their request? - How do they shape their lives after a denied request or granted (but not yet performed) request? - How do they view the relation between PAD and suicide?	

One researcher (RP) conducted all the interviews. Because of the situation around Covid-19, 4 interviews were held through video calling, but the remaining 17 could be held face to face, in conformity with the rules issued by the Dutch government. The researcher has a degree in Psychology and Philosophy. She had considerable experience with interviews from doing other studies. A psychiatrist from the project group with experience in evaluating PAD requests of persons suffering from mental illness was on standby for coaching RP. They, for example, discussed how to ask questions, how to handle suicidal remarks, or how to deal with an emotional respondent.

This study was evaluated and approved by the medical ethics committee (METC) of the Amsterdam UMC. To create conditions that were both safe for the respondent and the researcher, we asked the respondents to choose a care provider whom we would inform of his/her participation. We only shared that the respondent was going to participate in the study, and that we would contact the care provider if needed. The content of the interview remained confidential. We emphasized that respondents could always contact the researcher in case this was needed. Some respondents did so after the interview, to tell the researcher that their request was granted. No follow-up of participants was performed after the interview. The interviews were held at the location of the patient’s choice and lasted between 30 min and 2 h. Patients signed an informed-consent form that included statements on confidentiality and the voluntary character of participation. The patients who were interviewed via video calling were read the informed-consent form and agreed verbally.

The protocol of this study was evaluated and approved by the METC of the Amsterdam UMC.

### Respondents

Respondents were selected through purposive sampling and were selected using these criteria: they were above 16 and had a wish for PAD because of the suffering from their mental illness. We wanted to yield a broad variety of experiences, so we did not select based on age or status of the request. We excluded patients who also suffered from somatic illness, or who were involuntarily admitted to a psychiatric hospital/facility.

After 21 interviews, data saturation was reached as the last interviews did not yield any new information, and the inclusion was stopped. We interviewed mostly females (Dees et al. 2011) who were between the ages of 50–70 years old (Rooney et al. 2017). The main diagnosis was a mood disorder, but many respondents had multiple psychiatric diagnoses. In addition to suffering from a mood disorder, respondents often suffered from traumatic events in their past.

Almost all respondents suffered from their mental illness for more than a decade (even respondents who were 30 years or younger). Most respondents were still in the process of evaluation of their PAD request (Lemmens 2016), but we also interviewed respondents with granted requests (2020) or with a denied request (Onwuteaka-Philipsen et al. 2017). Others were ‘on hold’ while still in treatment or were on the waiting list of Expertisecentre Euthanasia.

We recruited the respondents through patient federations and the Dutch Dying with Dignity Foundation (NVVE). The researchers wrote a call, to which potential respondents could respond; we did not actively approach potential respondents individually. The call was initiated by the Amsterdam UMC and only distributed by the NVVE and patient federations.

### Data Analysis

All interviews were transcribed verbatim by a third party, who had signed a confidentiality statement. One interview was lost due to technical problems, which means that 20 interviews were included in the analysis. Analysis of all interviews was done by RP, and methods and results (the code trees and themes that were identified) were discussed with the supervisors of the study (SV and DW). SV also reads two interviews, to check whether the interviews were undertaken correctly.

We carried out thematic analysis and coded inductively (codes were identified and code trees were created). Analysis was performed with the use of a qualitative coding program: MAXQDA2020.

## Results

PAD can take two forms in the Netherlands: ‘euthanasia’ and ‘physician-assisted suicide.’ Euthanasia means the administration of life-ending drugs by a physician at the patient’s request and physician-assisted suicide is when a physician prescribes and provides life-ending drugs to the patient, at their own request, to self-administer. In the interviews, we use the term ‘euthanasia’ for both, because this is the most commonly used term in the Netherlands.

We identified broadly four themes concerning the meaning of the possibility of PAD for persons suffering from mental illness. These themes were (1) autonomy and self-determination, (2) ending the suffering, (3) recognition, and (4) dignified end-of-life. We also asked respondents about how they experience suicidality concerning PAD. These themes will be discussed below.

### Autonomy and Self-Determination

I think it is really important that it is a possibility. What I said before, you did not choose to be here, so why can’t you decide yourself when to get out of life. ‘That’s not possible here’, what do you mean? You can choose about everything yourself, but not that? And yes, what I said, I am very much in favor of being able to do that. Not only for me, some people are even worse off than me. R5 (request status: ‘on hold’)

Respondents stressed that they consider it important to be able to make decisions about their end of life. They indicated that they wanted to decide whether or not they die, and when and under what conditions their death should take place. Some considered themselves to be constantly living for others and, therefore, found self-determination even more important:

I’m very busy with that in my mind, that in this, others are not as important as I am. All my life I have been taking others into account, whether they liked it or not, whether it turned out well or not, half of it is meant well but does not come across like that. But I do have the feeling that…that I can choose myself in this. R3 (request status: ‘denied’)

### Ending the Suffering

And I thought about whether to apply [i.e. for PAD] for months, because I have wanted to die all my life. I have so much sadness, and am in so much pain, I cannot take it anymore, I am tired, exhausted, I have to fight every day so hard to get through the day, to get to the next day. I am tired, I do not want to do this anymore. R9 (request status: ‘has not made official request yet’)

Although it was stated by some respondents that they just wanted to die, most respondents indicated that they did not necessarily want to die, but that the life they live is just not bearable anymore. Respondents mentioned that they had been thinking about the decision to request PAD for a long time, and concluded that fighting their mental illness was just too much, and that they could not take it anymore. PAD was considered to be a way out of the pain and suffering that respondents experienced.

While some respondents wished to end their suffering as soon as possible, others considered PAD an option in case the suffering became even greater in the future. The option of PAD in case nothing works anymore would give peace of mind to the respondent and could potentially even help in their treatment.

R: I called the End-of-Life Clinic, (…) and they said that it [i.e. requesting PAD] was pointless, don’t even bother to try.

I: Okay, and how do you feel about that?

R: It is frustrating. I think, it would help me psychologically….in my treatment, psychologically it would help me a lot when it is always a possibility. In case it won’t work anymore, if you think it won’t work anymore. It always gave me a lot of peace of mind to find out, at the time, to find out how to kill myself, if I would. I believe it gives you a certain peace of mind to know you do not have to do it anymore in case it is really finished, if you believe that nothing works anymore. R11 (request status: ‘discusses request with GP’)

When asked what made the suffering so unbearable and impossible to deal with, respondents indicated that there was not just one reason; it was a convergence of reasons. Although the symptoms of the mental disorder played a role, respondents also mentioned other reasons that made their suffering unbearable. These reasons were often related to relations with other people; patients felt lonely, unable to feel a connection and/or have meaningful relations with other people:

R: And just, not wanting to do anything, nothing yields pleasure. And it has been the case, for a very long time, that I…well, no intimacy of whatsoever, that has never developed. I have been together with my partner for 27 years (…) but it does not come to me, I do not feel it. Also with friends, that is very difficult, if friends for example say to you that they like seeing me or talking to me, or do something together with me….I always had the feeling, from a young age, like ‘that is not true’, like it is not right, that they rather spend their time with someone else. R8 (request status: ‘granted’)

In addition to this, respondents did not want to feel like a burden to others, which they sometimes did. Also, they felt as if their life was without meaning or goals, which left them feeling worthless and empty. Another important reason related to the unbearableness of the suffering was that they considered their suffering to be without an end. This respondent explains how the fact that the suffering has to go on for such a long time makes it unbearable.

Nothing is left, really, nothing. And I cannot live with that. Maybe that is different for someone else (…) but for me that is too much. (…) If you cannot develop yourself, cannot evolve, and the only thing is sadness and misery. (…) I just want that to end, I believe it is enough like this. And yes, to me it is always so weird that with euthanasia, not necessarily with mental suffering, but with most people that receive euthanasia, these are patients in the terminal stage of their disease, often cancer patients. And I always find that a bit lame, because they get to hear that they have 3 more weeks to live, and that is not without a prospect of improvement to me, because it is 3 weeks. If somebody would tell me that this would take 3 more weeks, I would easily get through that. R21 (request status: ‘request is being evaluated by own psychiatrist’)

### Recognition

Some respondents acknowledged that even though they did have a wish to die, their request for PAD was meant as a ‘cry for help,’ they wanted to be seen and heard, as they felt that no one did so.

R: ‘I think, I see now, in hindsight that the euthanasia request was some sort of cry for help. ‘Up to here, and not further’. ‘And please do something, listen to me about what is going on’. So I can get on for a while, until that wish appears again. I mean subliminal, under the surface’

I: Latently

R: Yes, it is latently present R17 (request status: ‘discusses request with GP’)

Recognition was thought of in two different ways: recognition of their situation and their suffering, and recognition in the form of an affirmation by the physician that their suffering is indeed unbearable and without a prospect of improvement. Recognition of their suffering and wish to die as a result of that suffering was of utmost importance to some respondents. This perceived recognition gave them peace of mind, which paradoxically could create the possibility of not wanting PAD anymore. Recognition was considered not only important to the patient himself, but also for their environment.

So it would, that is the most important thing to me, that it would offer me the possibility to, for the people who have distanced themselves from me because it got too complicated, to reassure them, like, ‘okay, it goes along the official ways’. ‘Apparently, there is nothing we can do for her anymore’. (…) But if doctors say so and want to guide me through it, then it is not only me who is saying it, but also the expert. That is an important back-up that I have, so that it can happen in a normal way. That would be the nicest for me, yes. R21 (request status: ‘request is being evaluated by own psychiatrist’)

They explained that they considered that they might have a ‘tunnel vision’ about their own situation, and to have a physician to ‘objectively’ evaluate their situation meant that they could view their suffering as indeed unbearable and without a prospect of improvement.

I: Okay, and what does it mean to you to be able to make an euthanasia request?

R: Well, it gave me quite some peace of mind, because, well, that is what I feel, that it is nice to have some sort of approval. That you, well, because you have been in the midst of it for such a long time, and that causes you not to be objective anymore, with regard to the unbearableness and irremediableness of it. And it is nice that they can check independently whether that is correct or not. Because if you decide it all on your own, then you are stuck with the feeling of ‘that is how I decided it, and I believe it is unbearable and irremediable, but perhaps it is not’. And that approval, I think for me is very important. Maybe not for others, but I think that is part of my character. So it might not be the case for others, but for me it is very nice to receive that confirmation. R7 (request status: ‘granted’)

Many respondents indicated that they did not felt heard by the mental-healthcare system (GGZ) in the Netherlands; they felt that the quality of the GGZ was lacking. This respondent for example mentioned how she felt a victim of the mental-healthcare system:

R: When I was 17, I ended up in the mental healthcare system

I: How did you experience that, how did that work out for you?

R: Well, I would not recommend it to anyone to enter the mental healthcare system.

I: Okay…

R: I say this with a smile, but it has been one big nightmare. After ten years, the past ten years…well, the system is just, I feel a victim of it, to be honest, more than I was helped by them. With a sick management culture and jargon, and perverse incentives because of the power of the health insurer, nothing is right about the entire system. And people blundered with me, all those years. And I, especially since 2016, 2017, I had to say ‘listen to me finally, take me seriously!’ And that was a turning point for me, that I got the feeling like ‘you are not with me, you are my enemy now. R21 (request status: ‘request is being evaluated by own psychiatrist’)

Reasons for speaking negatively about the GGZ were the following: they did not feel as if the GGZ took responsibility in taking up a request for PAD, as the treating psychiatrist left this to EE. Treatment did not seem accessible for those with a wish for PAD and they felt they were denied therapy options because this was considered by the mental-healthcare professionals as incompatible with a wish for PAD. Others state that the GGZ focuses too much on curation and too little on personal recovery from the psychiatric condition. Others complained about the lack of continuity with regard to therapists that aftercare is not well taken care of, and that protocols are too rigid and that mental healthcare is not accessible enough.

Some respondents indicated that they did not want to die but just wanted to talk to the physician to feel heard by them. It was suggested that not feeling heard by mental-healthcare professionals can result in starting a conversation about PAD.

Yes, and maybe, maybe it is also….well, that I just want to talk to those people, maybe also to just feel heard. If you look deep into my heart, I do not want to die… and yes, the process can take up to a year. And you can always say ‘no’, but I just wanted to start the trajectory. (…) I just want to talk to those people. R1 (request status: ‘request status: ‘waiting list’

It seems as if in some cases, not feeling heard by mental-healthcare professionals can provoke the patient into a wish to discuss PAD. However, this does not mean that the patient is not suffering from their mental illness; it shows how important it is to the patient to feel recognized in their suffering by mental-healthcare professionals and what the patient is willing to consider and discuss to feel heard.

### Dignified End-of-Life

An important theme that we identified involved references to a ‘dignified’ or ‘humane’ end-of-life. Respondents provided us with different aspects which, to them, are essential to a ‘dignified’ or ‘humane’ end-of-life.

The first aspect of a dignified death was that PAD provided an ‘open and honest’ way of dying, involving others and without the risk of legal persecution for the relatives.

I believe it is a dignified way to say goodbye. (…) And that there are people that grant me to do it like this, with relatives, completely open, in a way that is 100% sure. That they won’t intervene, (…), that you don’t have to do secretly or illegally. That it can happen in such an open and honest way. Yes, that is a big difference with other ways. R8 (request status: ‘granted’)

A second aspect was the prevention of suicide. Suicide was perceived as insecure and inhumane, for both the patient and others. Not succeeding while trying to commit suicide, with all the possible consequences (for example, disability) was something that the patient wanted to avoid, for himself, and their loved ones.

But if it does not work out…(…) there are a lot of consequences, for myself, because I damage my body, or cannot do things anymore and life will become even more unbearable. You break things, cut things up that cannot heal anymore or will be painful, that you make you suffer even more. So that is a hard consideration. And, I don’t have to take that step with an euthanasia request(…) It doesn’t matter really how they kill me, as long as they do, and that the wish is accepted, that they don’t stop you. R19 (request status: ‘request is being evaluated by EE’)

### Suicide and PAD

As suicide was considered not to be a part of a dignified or humane death, many respondents spoke about how they wished to prevent this from happening. One of the reasons that physicians struggle with PAD for patients who suffer from mental illness, is that they have trouble distinguishing suicidality from a wish for PAD. Our respondents clearly distinguished ‘impulsive suicidality’ from PAD. They recognized that impulsive suicidality was part of their psychopathology.

R: But the attempts were in a period when I was depressed

I: Okay, and was it the sombreness that made you suicidal?

R: Yes, and the voices. The orders and not wanting life anymore.

I: So the voices gave you the order to kill yourself?

R: Yes, they have only one goal: to destroy me, they are only negative. R1 (request status: ‘waiting list’)

Respondents experienced impulsive suicidality as different from a request for PAD; suicidality, although sometimes also planned, was perceived as an act out of desperation and crisis; a state of mind in which there is no more room for other thoughts or control over actions. A wish for PAD was seen to be more well considered.

Being suicidal is often despair and panic. And of course that you want it, but at that moment when you have those thoughts and want to carry it out, or think about that, then you are at a low. While with a euthanasia request, of course you also have those moments, it can be any moment, it can happen any time, but the euthanasia trajectory is very long, and there are moments that you are not suicidal anymore, that you are just left with ‘I am done with life, I want the suffering to end, and to die’ That can be a difference with the euthanasia trajectory. R19 (request status: request is being evaluated by EE’)

In addition to the decision-making process being more well considered in the case of PAD, another distinguishing factor identified was that PAD was perceived as more humane than suicide, because the way that the death wish was carried out was more ‘friendly,’ also for others that otherwise may be confronted with a gruesome suicide.

R: Most of the time, suicide is very gruesome. And euthanasia is possible, but from my perspective it is so much more respectful and friendly. So, there is a difference, being suicidal, and ending your life is different from euthanasia.

I: And is the main difference then the way it is performed, like, suicide is gruesome and euthanasia can be peaceful?

R: Yes, a humane manner. A quiet, humane, loving and respectful way. R20 (request status: request is being evaluated by EE’)

A final difference between suicide and PAD mentioned was that a request for PAD comes more ‘from the core’ than suicidality does.

4.13 I: How do you see that relation? How does that work for you? Is it the same, or different?

R: Suicide is different from a wish to die, I think. Suicidality tends towards crisis and panic, and a wish to die is more ‘deep’.

I: Okay, and with ‘deep’ you mean?

R: From your true core, that you cannot take it anymore…the wish to die.

I: Yes.

R: And suicidality can be more random. R13 (request status: ‘granted’)

Also, the term ‘self-euthanasia’ was also mentioned, as a sort of intermediate form between impulsive suicidality and PAD. ‘Self-euthanasia’ was not only considered a less impulsive form of suicide but had more distinctive elements to it: there is no physician involved who has to decide over the patient’s fate, self-euthanasia can be carried out when the patient wants it, one can involve relatives and loved ones, and it is less aggressive than suicide as it is carried out with, for example, medication or the helium method instead of jumping in front of a train or off a high-rise building. Other respondents did not consider self-euthanasia a proper alternative to PAD, as it is not sure whether one will definitely die from self-euthanasia; there is the risk of failure, and relatives may be confronted with legal issues.

What I see as a difference for myself, because I saw this also in my surroundings, in therapy and also in work, is the acute impulsive suicidality, taking action impulsively, and that is not the case with me. My brain does not work like that. With me, it would always be a planned, well-prepared action. So in that sense, and that is a term that I found at the NVVE [i.e. Dutch Dying with Dignity Foundation], it would be more like a ‘self-euthanasia’ than a suicide, in an impulsive form that you see a lot when people tried multiple times. That is not the case with me. (…). For me, that is the difference. So there would be a suicide wish in the sense that I rather die today than tomorrow, but I would never leave impulsively and leave everyone with the misery that brings along. R8 (request status: ‘granted’)

## Discussion

We conducted this study to gain insight into the question of what the possibility of PAD means to patients suffering from mental illness who have a wish to die. We identified four themes: autonomy and self-determination, ending of the suffering, recognition, and a dignified end of life. The respondents considered it very important that they can choose a humane death, to end their suffering or to prevent a gruesome suicide or an unsuccessful suicide attempt that may cause disability. Although not all patients necessarily wish to die, the fact that their suffering is so unbearable and does not seem to have a foreseeable end is too much for them to handle. They choose death over living their lives like this. The unbearableness of the suffering has several elements that altogether make it unbearable to the patient: suffering from the symptoms of the mental illness, not having satisfying relationships, difficulty giving meaning to life, and perceived endlessness of the suffering. Verhofstadt et al. found the same domains (medical, intrapersonal, interpersonal, societal, and existential) in their study on the unbearableness of suffering of patients with mental illness and a request for PAD in Belgium (Verhofstadt et al. 2017). Dees et al. found that also in the case of somatic suffering, existential or psycho-emotional components contributed significantly to the unbearableness (Dees et al. 2011). This shows that regardless the condition that people suffer from (mental or somatic), unbearableness is a complex phenomenon. Health care primarily focuses on symptom reduction, but if we take into account that unbearableness is not only determined by medical issues, one can question whether we should turn to healthcare in order to solve these issues?

We also showed that patients make a clear distinction between suicidality and PAD; although both revolve around wanting to die, suicidality is recognized, also by them, as being a part of the psychopathology and as a state of despair. From the perspective of the patients, the difficulty in differentiating between suicidality and a request for PAD seems to be a theoretical problem and not a problem that they recognize or experience (Kim et al. 2018).

What is remarkable is that across the themes we identified, ‘peace of mind’ plays a significant role in relation to PAD. For some, the possibility of PAD in the future is enough to make it possible for them to continue living for that moment (and it may even help them in therapy). Thienpont et al. already showed that a significant number of patients continue living after they received approval from their physician to receive PAD (Thienpont et al. 2015). We now established that not only a granted request may bring in a new perspective that helps to continue living, but that the presence of the option of PAD in itself already creates peace of mind, and that it may even prevent suicide because it helps patients in their treatment and to keep their mind off of suicide. Having the option of PAD and being able to discuss this with a physician are, thus, very important to the patient. We want to highlight this point, as it has been argued that physicians should not discuss PAD with patients as this would take away hope from the patient (Blikshavn et al. 2017; Berghmans et al. 2013). Patients say that they benefit from discussing their wish to die and PAD request with their physician. This is also reflected in the fact that patients attach importance to ‘recognition.’ Respondents feel acknowledged if the physician takes their request for PAD seriously and discusses it with them, which possibly could lead to new perspectives. This provides a strong argument in favor of discussing PAD with a patient. In our view, discussing a death wish with a patient should be a part of treatment, even if the physician him/herself does not want to perform the PAD. The treating physician knows the patient best. In addition, being able to talk about your inner world and wishes is an essential part of psychological treatment. It is problematic if a patient cannot talk about their wish, because the physician is not open to it; it ignores a big part of the patient’s life and longing. Also, talking about the death wish can open up the possibility of new perspectives on treatment. If the patient is assured that his/her wish is taken seriously, our study shows that the patient can experience enough ‘peace of mind’ to think about new possibilities. This could increase the willingness of the patient to try out new treatment options that could lead to an improvement of their situation.

Remarkably, respondents mentioned that they wished to receive this recognition specifically from their mental-healthcare provider, and not for example from their spouses, friends, or other important persons in their lives. This study could not answer the question of whether they did or did not wish for recognition from their immediate surroundings, or whether they already did. Fact is, they do explicitly mention this wish in relation to their mental-healthcare provider. It could be argued that acknowledging a patient’s suffering is always part of a good treatment. The question remains whether a ‘formal’ discussion about PAD is the right or only way to provide it. Maybe, if mental-healthcare providers take death wishes seriously in an earlier phase of treatment and are willing to discuss these, patients do not need to ask for PAD in order to receive that recognition.

In our view, mental healthcare in the Netherlands (in collaboration with patients) needs to reflect on how to better meet the needs of the patient. Recognition of their suffering and discussing their death wish in an earlier phase of treatment could perhaps prevent patients with mental illnesses from considering PAD.

## Conclusion

We believe that the main take-home message from this study is that it is beneficial to patients to discuss their wish for PAD with a physician, because it provides them with recognition and may even cause them to not want to die anymore. We would suggest that the patient’s own treating physician takes up this role. It seems many of our respondents only were able to discuss their death wish with a physician from EE. Discussing a death wish should be part of treatment, even if the physician decides he/she does not want to perform a PAD request

### Strengths and Limitations

One of the strengths of this study is that we included the voice of the people that this debate revolves around: patients that suffer from mental illnesses and who have a wish to die. It would be paternalistic to only talk about the patient, and not *with* the patient. Persons with mental illness are often excluded from debates as they are perceived to be especially vulnerable or not capable to discuss their experiences. Although we took precautionary measures in order to provide safety for both the patient and the researcher, partly on our own initiative, partly on request of the medical ethics committee, these were not needed during or after the interviews.

The general response from the respondents was that they finally had a voice, that they were listened to, that they found meaning in being listened to, and that it was important to do this research. One respondent even indicated that because of the interview, she could find meaning again in her life and wanted to do something with her experience as a person who has a mental illness; her story had worth.

A limitation of this study is that it could be the case that respondents who responded to our call are patients who are disproportionality dissatisfied with the mental-healthcare system in the Netherlands and wanted to talk to us to express that dissatisfaction. This could have led to a bias in our results. Another limitation is that the coding was performed by only one researcher (RP). Although she has considerable experience with interviewing and coding from other studies, this could have led to a bias in our results. We limited this by discussing code trees and themes with both supervisors. Also, two transcripts of interviews were read by one supervisor (SV).

The variation in the patients’ phases of decision making with regard to their PAD wish could be seen as a limitation to the study. We recruited respondents in various stages of their trajectory for the following reason: there are not that many patients with mental illness and a PAD wish who also want to share their experiences, and this has provided us with the opportunity to include a substantial amount of respondents. In addition, although they differ in phase, their wishes remain the same, namely to receive PAD because they suffered from a mental illness. A final limitation could be that we did not ask for marital status, occupation, educational level, or other characteristics, which could complicate the replication of the study.
